# Accelerated AGEing: The Impact of Advanced Glycation End Products on the Prognosis of Chronic Kidney Disease

**DOI:** 10.3390/antiox12030584

**Published:** 2023-02-26

**Authors:** Elena Dozio, Lara Caldiroli, Paolo Molinari, Giuseppe Castellano, Nicholas Walter Delfrate, Massimiliano Marco Corsi Romanelli, Simone Vettoretti

**Affiliations:** 1 Department of Biomedical Science for Health, Università degli Studi di Milano, 20133 Milan, Italy; 2 Unit of Nephrology, Dialysis and Kidney Transplantation, Fondazione IRCCS Ca’ Granda Ospedale Maggiore Policlinico di Milano, 20122 Milan, Italy; 3 Department of Clinical Sciences and Community Health, Università degli Studi di Milano, 20122 Milan, Italy; 4 Service of Laboratory Medicine1-Clinical Pathology, IRCCS Policlinico San Donato, San Donato Milanese, 20097 Milan, Italy

**Keywords:** advanced glycation end products (AGEs), aging, cardiovascular diseases, chronic kidney disease (CKD), inflammation, mortality, pro-oxidant milieu, sarcopenia

## Abstract

Advanced glycation end products (AGEs) are aging products. In chronic kidney disease (CKD), AGEs accumulate due to the increased production, reduced excretion, and the imbalance between oxidant/antioxidant capacities. CKD is therefore a model of aging. The aim of this review is to summarize the present knowledge of AGEs in CKD onset and progression, also focusing on CKD-related disorders (cardiovascular diseases, sarcopenia, and nutritional imbalance) and CKD mortality. The role of AGEs as etiopathogenetic molecules, as well as potential markers of disease progression and/or therapeutic targets, will be discussed.

## 1. Introduction

Advanced glycation end products (AGEs) are known as aging products. In chronic kidney disease (CKD), AGEs build up due to increased production, reduced excretion, and imbalance between oxidizing/antioxidant abilities. CKD is therefore a model of aging. AGEs can, in turn, promote CKD progression and CKD-related complications. AGEs are studied not only for their etiopathogenetic role but also as new potential markers of disease progression and/or therapeutic targets. The aim of this review is to summarize the present knowledge of AGEs in CKD and CKD-related disorders.

## 2. Advanced Glycation End Products (AGEs) in Chronic Kidney Disease (CKD): Synthesis and Endogenous Defensive Strategies

AGEs are a class of compounds derived from the non-enzymatic reaction of glucose and its metabolites with protein, lipids, and nucleic acids. Since hyperglycemia is the main promoter of AGE synthesis, the highest concentrations of AGEs can be observed in diabetes mellitus (DM). Aging and other pathological conditions characterized by increased oxidative stress and inflammation may promote AGE accumulation through the synthesis of highly reactive carbonyl intermediates and their interaction with other bioactive molecules [1,2].

**Figure 1:**
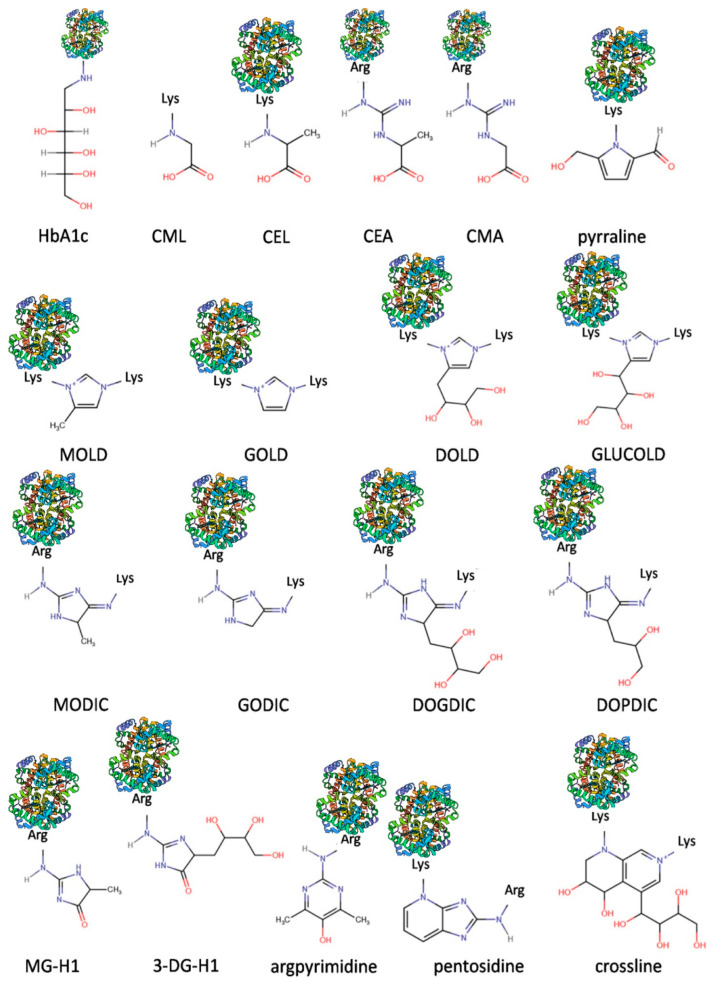
Different types of AGE–protein compounds. **Note:** Glycated hemoglobin (HbA1c), N^ε^–(carboxymethyl)lysine (CML), N^ε^-(1-carboxyethyl)lysine (CEL), N^7^–(1-carboxyethyl)arginine (CEA), N^7^–(carboxymethyl)arginine (CMA), 6-(2-formyl-5-hydroxymethyl-1-pyrrolyl)-L-norleucine (pyrraline), 6-{1-[(5S)-5-ammonio-6-oxido-6-oxohexyl]-4-methyl-imidazolium-3-yl}-L-norleucine (MOLD), 6-{1-[(5S)-5-ammonio-6-oxido-6-oxohexyl]imidazolium-3-yl}-L-norleucine (GOLD),1,3-di(N^ε^-lysino)-4-(2,3,4-trihydroxybutyl)-imidazolium (DOLD),1,3-bis-(5-amino-5-carboxypentyl)-4-(1,2,3,4-tetrahydroxybutyl)-3H-imidazolium (GLUCOLD), 2-ammonio-6-({2-[4-ammonio-5-oxido-5-oxopently)amino]4-methyl-4,5-dihydro-1H-imidazol-5-ylidene}amino)hexanoate (MODIC), N^6^-(2-{4S(-4-ammonio-5-oxido-5-oxopentyl]amino}-3,5-dihydro-4H-imidazol-4-ylidene)-L-lysine (GODIC), N^6^-{2-{[(4S)-4-ammonio-5-oxido-5-oxopentyl]amino}-5-[(2S,3R)-2,3,4-trihydroxybutyl]-3,5-dihydro-4H-imidazol-4-ylidene}-L-lysinate (DOGDIC), N^6^-{2-{[(4S)-4-ammonio-5-oxido-5-oxopentyl]amino}-5-[(2S)-2,3-dixydroxypropyl]3,5-dihydro-4H-imidazol-4-ylidene}-L-lysinate (DOPDIC), N^δ^-(5-methyl-4-imidazolon-2-yl)-L-ornithine (MG-H1), N^δ^-(45-hydro-5-(2,3,4-trihydroxybutyl)-4-imidazolon-2-yl]-L-ornithine (3DG-H1), N^δ^-(5-hydroxy-4,6-dimethylpyrimidine-2-yl)-L-ornithine (argpyrimidine), and 6-[2-[[(4S)-4-amino-5-hydroxy-5-oxopentyl]amino]-4-imidazo [4,5-b]pyridinyl]-L-norleucine (pentosidine). Modified protein surface models are based on the structure of human hemoglobin (PDB ID 1COH).

The Maillard reaction is one of the main pathways leading to AGE synthesis. This is a series of non-enzymatic reactions in which the carbonyl groups of reducing sugars, such as glucose, react with amino groups to produce Schiff bases, which then undergo Amadori rearrangements and further oxidative modifications (glycoxidation). These reactions are promoted by reactive oxygen species (ROS) and reactive nitrogen species (RNS) and give rise to dicarbonyl compounds such as glyoxal. Auto-oxidation of glucose and Schiff bases are other sources of dicarbonyl compounds, such as methylglyoxal, which can further react with amino groups and undergo further glycoxidation. The result is a variety of protein cross links and protein modifications known as AGEs. Lysine and arginine residues are the main target amino acids involved in the synthesis of AGEs. From a chemical point of view, the AGE family is very heterogeneous and includes fluorescent cross-linking AGEs (i.e., vesperlysine, pentosidine, and crossline), non-fluorescent cross-linking AGEs (i.e., imidazolium dilysine cross links, alkyl formyl glycosyl pyrroles, and arginine–lysine imidazole cross links), and non-cross-linking AGEs (i.e., N-fructosyl-lysine, N carboxyethyl-lysine, and N-carboxymethyl-lysine) [1,2,3,4]. AGE production is a relatively slow mechanism under physiological conditions, but once formed, the process is nearly irreversible. Different defensive strategies to prevent their synthesis and accumulation exist, but aging and the pro-oxidant and proinflammatory milieu that characterizes many chronic disorders such as CKD induces an imbalance between AGE synthesis and detoxification. The main AGE compounds of biologic importance are represented in Figure 1.

Oxidative stress promotes the accumulation of reactive carbonyl compounds, which are AGE precursors. Different enzymes, such as aldose reductase, aldehyde dehydrogenase, and glyoxalase-1 and -2 (GLO-1 and GLO-2), can work as first-line defensive strategies, preventing AGE synthesis. GLO-1 and GLO-2 block AGE synthesis by promoting the degradation of dicarbonyl compounds [5]. GLO-1, which catalyzes the first step of detoxification, is the rate-limiting enzyme, and its activity strongly depends on glutathione concentration. Its function is affected both by gene expression and post-translational modifications.

Other membrane receptors are involved in AGE metabolism. These receptors include RAGE (receptor for AGEs), AGE-R1 (OST-48, p60), AGE-R2 (80K-H phosphoprotein), AGE-R3 (galectin-3), and the macrophage scavenger receptors ScR-II and CD-36 [6]. Except for RAGE, the other molecules are clearance receptors; they remove AGEs from the circulation and promote their degradation. AGE-R1, AGE-R2, and AGE-R3 are components of the AGE–receptor complex. This receptor is mainly localized in the caveolin-rich membrane domain and, by taking up AGEs, promotes their degradation and prevents AGE-damaging effects [7]. AGE-R1 was the first discovered and the most expressed of these components. Its level increases in parallel with AGEs, but it is downregulated by persistently high levels of AGEs [8]. AGE-R1 promotes AGE turnover by mesangial cells and contributes to counter regulation of AGE-induced inflammation [9]. AGE-R2 is a membrane protein without binding activities. It is involved in stabilization of the receptor complex in combination with AGE-R3, a carbohydrate-binding protein that shows strong affinity for a wide variety of AGEs and regulates different functions, from the cell cycle to inflammation [10,11]. Of interest, prolonged exposure to AGEs increases AGE-R3 expression [12].

**Figure 2:**
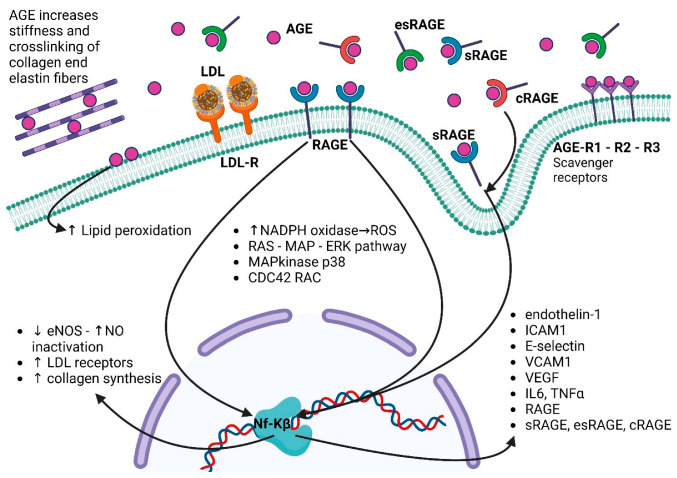
AGE–RAGE interaction at the intracellular level and RAGE activation cascade. **Note:** In the ECM, AGEs form on several different molecules, including lipids, collagen, laminin, elastin, and vitronectin. The formation of AGEs on ECM molecules impairs the constitution of the matrix and increases its stiffness. AGEs that bind to RAGEs on the cell surface begin a signaling cascade, stimulating NAD(P)H oxidase and increasing ROS, p21 RAS, and MAPKs. The ligand–RAGE interaction may also stimulate signaling via p38 MAPK and Rac/Cdc. A key target of RAGE signaling is Nf-κB. Nf-κB is translocated to the nucleus, where it promotes transcription of several proteins, including endothelin-1, ICAM-1, E-selectin, and tissue factor. Moreover AGE–RAGE interaction stimulates the synthesis of sRAGE (soluble receptor for advanced glycation end products), cRAGE (cleaved receptor of advanced glycation end products), and esRAGE (endogenous spliced receptor of advanced glycation end products). AGE and ligands for RAGE, sRAGE, and cRAGE trigger inflammatory response, while esRAGE serves as a scavenger molecule for circulating AGEs. AGEs seem to lower NO availability by decreasing activity of NOS and by quenching NO.

RAGE is a surface molecule that belongs to the immunoglobulin superfamily. It is a multiligand receptor that can recognize, bind, and transduce the signal of various molecules involved in the activation of the inflammatory response, both in homeostasis and in the presence of chronic diseases. The transduction of the RAGE-mediated signal leads to the formation of ROS, activation of the transcription factor NF-kB (nuclear factor kappa-light-chain-enhancer of activated B cells), and the upregulation of inflammatory mediators and adhesion molecules. AGEs, S100 calgranulins, β-amyloid peptides, and amphoterins are among the main RAGE ligands. The RAGE gene extends for 3080 bp in humans and is located on chromosome 6 within the HLA class III near the junction area with class II. RAGE is transcribed at low levels in numerous cells. RAGE activation increases the expression of the receptor itself, with the consequent amplification of the response [13]. Along with the form of the membrane receptor, RAGE also exists as a soluble receptor (sRAGE). sRAGE includes the extracellular portion of the membrane form; it can bind the ligands, but it cannot transduce the signal. sRAGE is not a single form but a pool composed of two different isoforms: cRAGE (cleaved RAGE) and esRAGE (endogenous secretory RAGE). cRAGE is the result of the proteolytic cleavage of the membrane receptor promoted by different proteases, such as ADAM10 (a disintegrin and metalloproteinase 10) and MMP (metalloproteinase)-9, MMP-13, and MMP-3 [14,15]. In contrast, esRAGE derives from alternative gene splicing. Different splicing variants of RAGE exist, but, except for esRAGE, all are degraded at the mRNA level by the NMD (nonsense-mediated mRNA decay) pathway. esRAGE retains part of intron 9, with partial removal of exon 10. The result is a change in the reading sequence of the protein at amino acid 332 and the consequent loss of both the cytoplasmic and transmembrane domains. The resulting protein resolves at 48 kDa in size compared to 55 kDa for the membrane form [16]. sRAGE is a decoy receptor. By binding AGEs in the circulation, both cRAGE and esRAGE can prevent membrane RAGE activation and RAGE-induced proinflammatory and detrimental effects. Although the role is the same, the mechanisms promoting cRAGE and esRAGE synthesis are different. Therefore, we can suppose a different role as biomarkers for these two receptor forms. Because the expression and activity of the MMP enzymes that are involved in cRAGE synthesis are increased in inflammatory disorders, cRAGE can be considered a surrogate marker of inflammation. In contrast, esRAGE is considered the real decoy receptor that is produced as an endogenous defensive strategy against AGEs, i.e., sRAGE and esRAGE decrease in patients with hypertension [17] and metabolic syndrome (MS) [18]. All these biological processes are summarized in Figure 2.

Lower total sRAGE levels are also associated with increased body mass index (BMI), waist circumference (WC), and fat mass [19,20]. In contrast, in DM and CKD. sRAGE levels increase [21,22] have been independently associated with cardiovascular outcome [21,23]. This means that changes in sRAGE levels depend on the clinical setting. Simultaneous measurement of esRAGE and cRAGE could help to identify the contribution of each form to total sRAGE and their relationship with the disorder.

The kidney plays a major role in AGE metabolism and excretion. In healthy subjects, AGEs are filtered by the glomeruli and taken up in the proximal tubules or directly collected from the blood in the peritubular capillaries. AGE metabolism mainly occurs in the proximal tubule cells (PTCs). AGEs are then metabolized to non-reactive compounds that can be excreted into urine. PTCs express specific endocytotic receptors on their apical membrane, which take up and metabolize low-molecular-weight proteins (LMWPs) filtered by the glomeruli in the lysosomes. Megalin is a large glycoprotein (~600 kDa) of the low-density lipoprotein receptor family [24] known to serve as a major receptor for endocytosis of multiple LMWPs, including transcobalamin-B12, vitamin D-binding protein, retinol-binding protein, parathyroid hormone, insulin, β2-microglobulin, epidermal growth factor, prolactin, lysozyme, cytochrome c, α1-microglobulin, and transthyretin [25]. Megalin is also one of the receptors involved in AGE uptake and metabolism [26]. By using a rat yolk-sac-derived L2 cell line system and antimegalin IgG antibodies, it has been found that megalin is involved in AGE endocytosis through co-operation with other membrane and intracellular proteins and is then recycled to the cell membrane after endocytosis has occurred [25,27]. Cubilin and Na+–H+ exchanger 3 (NHE3) are two membrane proteins that interact with megalin and comodulate its endocytic function [28,29]. ANKRA (ankyrin-repeat family A protein), ARH (autosomal-recessive hypercholesterolemia), Dab2 (disabled protein 2), GIPC (Gα-interacting protein (GAIP) interacting protein C terminus), MAGI-1 (membrane-associated guanylate kinase with inverted orientation-1), and MegBP (megalin-binding protein) bind the cytoplasmic domain of megalin and are supposed to have signaling functions, leading to AGE metabolism and further excretion into the urine [30].

Among different conditions promoting AGE accumulation, CKD deserves great attention. With decreasing renal function, AGEs increase, regardless of the presence of DM [31]. Of interest, AGE accumulation can be observed not only in patients with already established kidney failure but also in individuals with or without DM, in whom reduced renal function is not yet clinically evident [32,33,34]. AGE levels are elevated in CKD due both to increased synthesis and reduced kidney filtration and tubular metabolism.

In CKD patients, uremic toxins and inflammatory mediators reduce the availability of detoxifying enzymes and the clearance of these products, thereby increasing carbonyl stress and AGE synthesis [8,26]. As better described in the next section, the increased concentration of AGEs can induce kidney damage at both the glomerular and tubular levels. Excess AGE content can lead to oxidative stress, inflammation, and lipid deposition in renal tubules [35,36,37]. The urinary level of full-length megalin was positively correlated with kidney damage in patients with DM. In cultured immortalized rat proximal tubule cells, AGE-modified BSA promoted lysosomal dysfunction and megalin excretion via extracellular vesicles [35]. AGE overload therefore leads to the progressive loss of tubular metabolism. With reduced megalin, AGE metabolism is impaired, and RAGE activation by AGEs in proximal tubular cells can further promote kidney damage [38], as well as at glomerular levels [39]. This mechanism, along with direct AGE-induced glomerular damage, contribute to an increase in circulating AGEs.

## 3. AGEs in CKD Onset and Progression

In CKD, AGEs accumulate because of both the reduced filtration by the kidneys and the increase in production due to an unbalanced oxidative/antioxidant metabolism. The uremic environment, which is characterized by increased oxidative stress and inflammation, supports the production of AGEs [40,41]. AGEs promote a worsening in kidney function [42] and the increase in cardiovascular risk and mortality in end-stage renal disease (ESRD) and in kidney-transplanted patients (RTx-ps) [40,42,43]. With the impairment of renal function, AGEs accumulate, and there is an amplification of the oxidative stress.

Studies based on murine models support the detrimental role of AGEs in kidney damage development, showing a thickening of the basement membrane and expansion of the mesangial matrix in AGE-injected animals [44]. This could be linked to an increase in TGFβ (transforming growth factor β) levels and a reduction in the production of nitric oxide consequent to AGE activities at the renal level [45]. Another cause of the noxious effects of AGEs may also be the activation of RAGE.

Anatomically, proximal renal tubule cells are characterized by a great number of lysosomes for protein absorption/degradation, and lots of mitochondria are needed to supply the energy needed to sustain their metabolism [37,46]. Most of substances filtered at the glomerular level are reabsorbed by proximal renal tubules through endocytosis. As reported above, proximal tubular endosomal–lysosomal activity is mainly controlled by megalin, an endocytic receptor [30,38,47]. The excessive uptake of AGEs causes proximal dysfunction of tubular lysosomes and it has been suggested to be involved in the development of DM nephropathy. AGEs that are reabsorbed by proximal renal tubules lead to increased glucotoxicity on PTCs [38,48,49]. Under severe hyperglycemia, continuous activation of the AGE–RAGE pathway results in a positive feedback loop that increases injury at the proximal tubular level in patients with DM nephropathy [38].

## 4. AGEs as Biomarkers of Mortality in CKD

In patients with CKD, uremia, chronic inflammation, and oxidative stress can lead to an increase in ED production, which, in turn, promotes CKD-related complications and mortality [50,51,52]. Kidneys normally filter circulating AGEs, but it has been observed that the levels of these products increase both in DM and non-DM nephropathy [40,41]. Higher serum AGE concentrations may contribute to the worsening of renal function and the increase in cardiovascular risk and mortality both in conservative and replacement therapy patients [41,42,43].

Skin autofluorescence (SAF), which is a measure of AGE accumulation, has been linked with increased mortality risk in dialysis patients. A landmark study conducted in 109 patients receiving hemodialysis (HD) showed, for the first time, strong evidence that SAF is an independent risk factor for all-cause mortality in CKD [41,53], along with previous cardiovascular disease (CVD), serum albumin, and C-reactive protein (CRP). Numerous successive studies and a meta-analyses [54] of HD patients have confirmed that higher SAF could be an independent risk factor for all-cause mortality [55,56]. Similarly, SAF seems to be an independent risk factor for increased mortality in peritoneal dialysis (PD) patients [57,58]. In a study by McIntyre et al., SAF levels were found to be similar in PD and HD patients, even if years of dialysis were significantly lower in the PD cohort [59]. On the contrary, Oleniuc et al. showed higher SAF in HD patients [60]. Two other studies compared SAF levels in both PD and HD patients after adjustment for the years of dialysis and other potential confounders. They found higher SAF in PD patients [61,62]. The glucose present in peritoneal dialysis fluid may be a key factor that contributes to AGE accumulation during PD [53]. This has been confirmed by several studies that showed an association between the magnitude of glucose exposure and SAF [59,62,63]. A study performed in a mixed population of patients with stage 5 CKD not yet on dialysis (n = 130), PD (n = 93) and HD (n = 38) explored the association of SAF and the augmentation index (AIx), a measure of arterial stiffness, with all-cause and CV mortality in comparison to traditional risk factors assessed with the Framingham Risk Score (FRS) [64]. Both SAF and AIx independently predicted these outcomes. SAF remained significant after adjustment for the FRS but not after additional adjustment for the presence of CVD, CRP, and serum albumin [64]. For this reason, SAF was confirmed to be a risk factor for mortality, but traditional risk factors still accounted for most of the observed risk.

SAF levels have also been investigated in patients with earlier stages of CKD. In a large study conducted by Fraser et al., SAF was observed to be elevated in 1707 persons with stage 3CKD (mean estimated glomerular filtration (eGFR), −52.5 ± 10.4 mL/min/1.73 m^2^). After 3.6 years of follow-up, SAF was associated with increased all-cause mortality even after adjustment for age and sex, although after adjustment for multiple risk factors, the association was no longer statistically significant [65].

A rise in sRAGE levels has also been linked with a higher mortality risk in HD and PD patients [66,67,68]. Recently, our group reported an association between increased sRAGE levels and mortality in HD and PD patients [69]. For these reasons, glycated albumin, an early precursor of AGEs correlated with the overall prognosis of patients on dialysis [70], may affect the prognosis of advanced CKD patients [69].

## 5. AGE–RAGE Pathway in Cardiovascular System: Role in the Etiopathogenesis of Diseases and as Biomarkers

The risk of CVD is increased in patients with CKD. As CKD progresses, the incidence of CVD events, hospitalization, and death increases [71]. Data from the US Renal Data System (USRDS) (2020) indicate that the risk of CVD in CKD was higher than in non-CKD patients [72]. Among CKD patients, the prevalence of CVD was higher in patients with stages G4–5 (75.3%) compared to stage G3 (66.6%) and stage G1-2 (63.4%). Several CVDs, including left ventricular dysfunctions, cardiomyopathy, atherosclerosis, stroke, heart failure, peripheral artery disease, abdominal aortic aneurysm, and venous thrombosis, are associated with CKD and represent the most common causes of death in CKD patients. Traditional CVD risk factors such as hypertension, advanced age, dyslipidemia, and DM, which are frequent in CKD, can only partially clarify the high risk of diseases in CKD. Malnutrition, anemia, accumulation of uremic toxins, activation of fibroblast growth factor 23 pathway (FGF-23) oxidative stress, and inflammation are other “non-traditional” risk factors in CKD that must be considered in computing the overall likelihood of developing CVD [73]. Therefore, the need for other risk markers is compelling in these patients.

AGEs are known to be involved in the onset and progression of different CVDs, regardless of the presence of CKD [74,75]. As previously discussed, the uremic milieu, along with reduced kidney function, may play a pivotal role in AGE generation and accumulation. Therefore, AGEs must be considered in CKD both for their pathogenetic role and as disease biomarkers and therapeutic targets to prevent and delay CVD complications.

Left ventricular hypertrophy (LVH), which can lead to heart failure (HF), is one of the main cardiac manifestations in CKD [76,77]. AGEs are supposed to activate at least three different pathways that can lead to HF. First, AGEs can directly promote protein cross linking, which undermines matrix protein flexibility, increases rigidity, and may ultimately lead to heart dysfunction. Second, by binding to RAGEs, AGEs activate intracellular signals that upregulate profibrotic mediators such as transforming grow factor β (TGF- β). Third, AGEs induce a delay in calcium uptake, which increases the duration of the repolarization phase of cardiac contraction [78]. AGEs can also promote LVH and increase the risk of HF by inducing the expression of FGF23, a recently discovered hormone that regulates phosphate and vitamin D metabolism by the kidney and has been proposed as an additional causal factor in pathologic LVH [79]. Furthermore, both translational and human studies have indicated a role of AGEs in HF. Diastolic function was shown to correlate with AGEs in diabetic obese rats, and calcium concentration was reduced in the heart of transgenic mice overexpressing RAGE [80,81]. Most studies describing the association of AGEs with systolic and diastolic function in humans were performed in non-CKD patients or in ESRD [82,83]. Plasma AGEs, in particular N(epsilon)-(carboxymethyl)- lysine (CML) levels, were correlated with the severity of HF and predicted patient outcome, even after correction for known predictors of outcome in HF [83]. High serum levels of pentosidine, another AGE, were suggested as a prognostic element and a marker for risk stratification in HF patients [82]. The association found between skin AGEs and HF among participants in the cross-sectional, population-based Rotterdam study indicated that higher levels of AGEs are associated with the higher odds of HF [84]. Similar results were described for ESRD patients, in whom pentosidine levels correlated with alterations in heart geometry [85]. Instead of AGEs, other studies explored the role of sRAGE as a diagnostic and prognostic marker of HF, but results are conflicting, as in CKD. Lower circulating levels of sRAGE were independently associated with the development of HF in the Atherosclerosis Risk in Communities Study [86]. In some other studies, high sRAGE levels predicted poorer prognosis in patients with HF [87,88]. However as previously mentioned, this evidence is not unanimous [89]. Previous research from our group suggested a potential role for high sRAGE levels as a prognostic factor for mortality in ESRD patients displaying an active process of cardiac remodeling [67]. In contrast, a significant inverse relationship was found between sRAGE and left ventricular mass index and mean wall thickness in CKD patients [90]. Considering that sRAGE levels increase with worsening of kidney function and in DM, their role as a marker of cardiac remodeling may be hampered by the clinical setting of the patients. For this reason, additional studies performed on selected patient subgroups could help to explain the discrepancies observed so far.

CKD patients—even those with a very mild reduction in renal function—display arterial stiffness, a vascular biomarker associated with an independent risk of CVD [91,92]. The stiffening process and its consequences are the result of uremic toxin accumulation, which promotes vascular inflammation, endothelial dysfunction, and calcification. AGEs are contributors, among different toxins, of the development of uremic vasculopathy. AGEs induce arterial stiffness by reducing the expression and phosphorylation of endothelial nitric oxide synthases, which leads to endothelial dysfunction and activation of the proinflammatory response, which further leads to cross linking of the media collagen molecules and osteogenic differentiation of vascular smooth muscle cells [93]. Furthermore, AGEs have been correlated with markers of coronary artery calcifications in both CKD and ESRD patients [94,95].

As a direct consequence of what has been stated above, CKD patients also experience a high incidence of atherosclerotic disease. Low-density lipoprotein (LDL) oxidation may begin atherosclerotic lesion formation or speed up its development. One potential mechanism linking CKD and the development of atherosclerosis is the modification of LDL by AGEs. Exposure of LDL to human, serum-derived AGEs leads to the synthesis of AGE–LDL, which mimics classic LDL oxidation [96,97]. Accumulation of AGEs in coronary atheroma in diabetic ESRD patients was also demonstrated using an anti-AGE-specific antibody [98]. Little information is available about the relationship between AGEs and subclinical atherosclerosis during the early stages of CKD. A close relationship was found between skin autofluorescence (AF) and asymptomatic atheromatous disease. Furthermore, skin AF was negatively correlated with eGFR, suggesting that AGE-related vascular disease could be one of the factors by which AGEs hamper renal function [99].

## 6. AGEs, Sarcopenia, and Nutritional Status: Pathogenic Role and Biomarker of Muscle Wasting

Currently, most studies regarding how AGEs can influence muscle function and their role in the onset and progression of sarcopenia are conducted in DM, cancer, and aging [100,101]. In muscle, myoblasts differentiate into myocytes. Anything that can promote myoblast loss and myocytes dysfunction affects skeletal muscle mass and promotes sarcopenia. In a murine study, C57Bl/6j mice fed a high-fat and high-sugar diet and ob/ob mice fed a standard diet showed inflammation, oxidative stress, and accumulated AGEs in muscle fibers. This increase in AGE levels can cause myosteatosis, a reduction in muscle mass, and a decrease in mitochondrial efficiency and induce the transition of fast-to-low-speed muscle fibers [101,102,103]. At the same time, increased RAGE expression on the cellular membrane and the consequent activation of the SCAP (SREBP cleavage-activating protein)/SREBP (sterol regulatory element binding protein) lipogenic pathway could be a link between intracellular AGE accumulation and muscle wasting [104]. Moreover, as observed in in C2C12 myoblasts, AGEs can also inhibit myoblast differentiation and promote cellular death [105]. Both in murine and human cell lines, it has been observed that AGE concentration is also associated with a decrease in myofiber diameter and increased expression of MAFbx (muscle atrophy F-box), a protein of the ubiquitin proteasome pathway, which can induce the degradation of intracellular protein in skeletal muscle [105]. Interestingly, it has been shown that chronic activation/overexpression of RAGE affects muscle fiber atrophy and inflammation, while its absence seems to delay the loss of muscle mass and strength [101]. Moreover, in a murine model, it was demonstrated that pharmacologic RAGE inhibition can revert skeletal muscle alteration induced by aging, [106] suggesting that the negative effects of AGEs on myotube and myogenesis can be prevented by an AGE inhibitor [105] and that the AGE–RAGE pathway may have a crucial role in inducing myopathy.

Mitochondria are involved in many fundamental cellular processes of skeletal muscle, such as energy supply, calcium homeostasis, and regulation of apoptosis [106]. Many studies have already proven the role of mitochondria in the onset of sarcopenia. In CKD, there is an imbalance between ROS generation and detoxification. Reduced kidney filtration promotes AGE accumulation and the activation of an inflammaging circle that may exacerbate mitochondrial dysfunctions. Although modulation of the AGE/RAGE axis has been proposed as a valid strategy to enhance mitochondrial damage, the link between RAGE, mitochondria, and inflammation and their role in the onset of sarcopenia are poorly described, although they seem to play a major role in CKD [107].

Studies conducted in elderly people, as well as in patients with diabetes, have suggested that AGEs are inversely associated with muscle strength and mass [100,108]. A reduction in appendicular lean mass was correlated with levels of an AGE subtype, pentosidine, which was indicated as a possible biomarker for sarcopenia [100]. In older women urinary excretion of CML, another AGE subtype, was negatively associated with grip strength and suggested as a possible screening tool for sarcopenia [108]. In a recent study, Yabuuchi et al. showed an association between increased AGE levels in the gastrocnemius muscle of nephrectomized mice and muscle morphological anomalies, capillary rarefaction, and mitochondrial dysfunctions [102]. Moreover, they demonstrated that in frail patients, serum AGE levels were significantly increased, while in dialysis patients, AGEs were inversely associated with physical performance and physical activity. In these patients, AGE–aptamer treatment improved the pathological derangement of muscle structure and function. AGEs were found to be associated with slowness and weight loss, two components of the Fried frailty phenotype. In older community-dwelling adults, a similar association has been observed, confirming that AGEs can affect muscle function [109]. In PD patients, Fonseca et al. observed an association between AGE accumulation and lower muscle stiffness/density [109].

Several mechanisms have been suggested as mediators of the negative effects of AGEs. Among these possible mechanisms, RAGE activation and subsequent inflammation, malnutrition, endothelial dysfunction, and connective tissue protein stiffness might explain how AGEs can worsen muscle function. CKD is characterized, among other complications, by insulin resistance and loss of muscle mass. Defects in insulin signaling can contribute to the development of sarcopenia. Insulin is an anabolic hormone. Insulin resistance and reduced insulin levels are known to correlate with protein breakdown, while increased insulin levels stimulate protein synthesis [110]. AGEs could be a link between insulin resistance and loss of muscle mass. Mice fed a high-fat and AGE-rich diet showed a reduction in insulin sensitivity [111]. It is also important to remember that in both healthy individuals and CKD patients, the dietary content of AGEs may influence the systemic levels of AGEs.

Since AGEs were found to be inversely correlated with average metabolic equivalent of task (MET) [102], exercise was suggested as a potential strategy to reduce AGE burden and improve AGE-related dysfunctions in CKD patients. However, more studies are needed to clarify this hypothesis.

Chronic inflammation and oxidative stress lead to muscle proteolysis and hyporexia, two mechanisms that lead to the development of malnutrition and hypoproteinemia [112,113]. Malnutrition may, in turn, promote AGE formation because it sustains an inflammatory and oxidative milieu.

In ESRD patients, since AGEs can cause weight loss and protein catabolism and enhance energy expenditure [102,114], AGE levels have been associated with the prevalence of malnutrition [102,115]. In a recent study conducted in patients with end-stage kidney disease, Suliman et al. found that plasma pentosidine levels were associated with inflammation and the development of malnutrition [102,115]. Inflammatory status can influence nutritional status in several ways; TNFα lead to a catabolic state through the induction of anorexia, stimulating both protein degradation and inhibition of protein synthesis [116,117,118]. Thus, advanced CKD patients with a higher inflammatory status develop a negative protein balance due to an inhibition of anabolic protein synthesis and the production of acute-phase proteins [118,119]. In a study by our group, we did not find any significant difference in serum AGE concentrations between well-nourished patients and patients at risk of malnutrition or malnourished patients. Instead, we observed an association between malnutrition and increased levels of sRAGEs and esRAGEs. The absence of a direct correlation between AGEs and malnutrition may depend on the fact that our study assessed patients with advanced CKD not yet on dialysis who did not have such high AGE levels as those described in dialyzed patients. Moreover, the three groups of patients classified according to their nutritional status showed the same value of eGFR. This may have weakened the possible effect of renal dysfunction degree on AGE levels [120].

**Figure 3:**
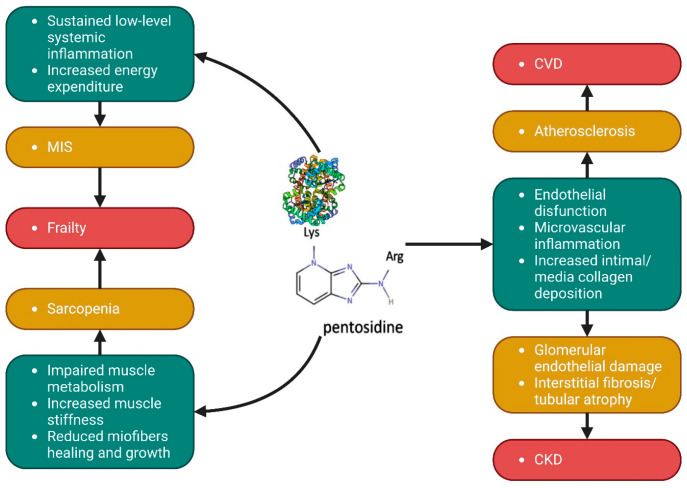
The influence of AGEs on nutrition, as well as muscle, cardiovascular, and kidney health. **Note:** MIS: malnutrition–inflammation syndrome; CVD: cardiovascular disease; CKD: chronic kidney disease. Modified protein surface models are based on the structure of human hemoglobin (PDB ID 1COH).

The main possible detrimental effects of AGEs on patient health are summarized in Figure 3.

## 7. Potential Future Therapeutic Solutions

Considering the role of the AGE–RAGE axis in CKD progression and involvement in CKD-related complications, this pathway could represent a potential treatment target. Therapeutic solutions could be aimed at reducing AGE intake, AGE formation and RAGE expression, blocking RAGE, and degrading AGEs and/or ROS.

1. AGE intake reduction: AGE levels can be lowered by reducing intake of food containing a high number of AGEs and encouraging consumption those containing the lowest amount of AGEs. AGE-rich foods include red meat, animal fat, cheese, sweetened food, and foods cooked at high temperatures in dry heat (frying, broiling, grilling, and roasting) [121]. This nutritional approach could be useful during the early stages of CKD, when patients are suggested which foods to consume and limit. In the advanced stage of CKD, patients are recommended to adhere more strictly to nutritional prescription. The reduced consumption of meat and animal-derived products is an ongoing practice in CKD patients that already limits AGE intake. Other AGE-lowering procedures include stopping cigarette smoking and exercise promotion [122,123].

Amelia K. Fotheringham et al. recently discussed the potential involvement of the gut microbiota in the modulation of plasma AGE concentration in CKD [124]. On one hand, it has been demonstrated that in dialysis patients, AGE restriction can modulate the microbiota [125]; however, we do not know how these changes can affect plasma concentration. Since the modulation of gut permeability and bacterial species results in a reduction in circulating inflammatory markers [126], we cannot exclude an effect on AGE synthesis and absorption, and further investigations are required.

2. Reduction of AGE synthesis: Several AGE-lowering compounds and AGE formation inhibitors have been developed and progressed to clinical trials; however, due to side effects or because companies have discontinued activities, these studies had to be retracted. AGE-lowering therapy can be classified into direct and indirect therapies. Names and mechanisms of action of molecules belonging to this group are presented in Table 1. Clinical trials of these anti-AGE drugs have been gradually developed. Despite some studies confirming some beneficial effects on AGE levels, nephropathy progression, endothelial function, inflammation, ventricular mass, and parameters of heart function, other reports produced conflicting results. A more detailed description of these studies and studies on other compounds can be found in the works by Sarmah S. at al. [127,128,129,130];
3. Suppression of RAGE expression: statins, candesartan, nifedipine, and rosiglitazone have been shown to reduce RAGE expression [131,132];
4. Blockade of RAGE activation.: Azeliragon has been used as a RAGE blocker in patients with Alzheimer’s disease, but it has not been applied to other clinal diseases [133]. Increasing sRAGE levels have also been proposed as an additional therapy. sRAGE levels can be increased by exogenous administration or by upregulating sRAGE expression with drugs such as statins, ACE inhibitors, and rosiglitazone [134,135,136]. sRAGE administration has been studied in preclinical models of atherosclerosis but not in humans [137]. sRAGE levels are already high in CKD, but since the AGE/sRAGE ratio still favors AGEs, this approach must not be excluded;
5. AGE degradation in vivo: GLO-1, the endogenous enzyme that blocks AGE synthesis by promoting the degradation of dicarbonyl compounds, is not available for clinical use, but its activity was upregulated in a placebo–control crossover clinical trial by the combined use of trans-resveratrol found in grapes and hesperidin found in orange [138];
6. Antioxidants: Transition metal ions are associated with the formation of AGEs and ROS generation. As a result, metal ion chelation can also prevent the formation of these products. In addition, antioxidants my reduce AGE–RAGE-induced oxidative stress [131,135].

**Table 1:** Direct and indirect AGE-lowering compounds.

Direct AGE-Lowering Compounds		
Name	Actions	Clinical Use/Development Stage
Algebrium chloride [139,140,141]	A carbonyl scavenger that can also reduce cross links and ROS.	Clinical trials demonstrated beneficial effects in treating cardiovascular disorders, but the company seems to have discontinued operations.
Aminoguanidine [142,143]	A scavenger of carbonyl and dicarbonyl compounds and an ROS inhibitor.	Investigated for the treatment of diabetic nephropathy. Clinical trials were started, but commercial efforts to develop aminoguanidine as a drug were stopped by the company.
Benfotiamine [144]	A synthetic, fat-soluble S-acyl derivative of thiamine (vitamin B1) that reduces glycolysis and the polyol pathway, ROS generation, and activation of transketolase.	Approved in some countries as a medication/dietary supplement to treat diabetic sensorimotor polyneuropathy.
Carnosine [145]	Scavenger of ROS and alpha-beta unsaturated aldehydes created by peroxidation of fatty acid cell membranes during oxidative stress. Carnosine can oppose glycation, and it can chelate divalent metal ions.	Available as a dietary supplement. Clinical trials suggested a renoprotective effect in diabetic nephropathy.
OPB-9195 [146]	A carbonyl scavenger that can also reduce ROS levels.	Used in preclinical studies; however, no human data on this agent have been published.
Piridoxamine [147]	It reacts with carbonyl groups in Amadori products, thereby preventing AGE synthesis from these intermediates. It can also scavenge and reduce ROS production by chelating transition metals.	In experimental models, reduced AGE accumulation by pyridoxamine improved renal function. Beneficial reduction from baseline in serum creatinine was demonstrated by clinical trials in diabetes.
Thiamine [148]	It reduces glycolysis and the polyol pathway, ROS generation, and activation of transketolase.	Beneficial reduction in urinary albumin excretion was demonstrated by clinical trials in diabetes.
**Direct AGE-Lowering Compounds**		
**Name**	**Actions**	**Clinical Use/Development Stage**
ACE inhibitors [149]	They inhibit ROS and, by reducing angiotensin II, exert anti-inflammatory effects.	Already used in clinical practice for hypertension. Ramipril reduced fluorescent AGEs, blood pressure, and proteinuria in diabetes.
Statins [150,151]	They stimulate RAGE shedding.	Already used in clinical practice for hypercholesterolemia. Simvastin reduced carotid plaque RAGE expression by decreasing AGE generation in diabetes. Atorvastatin reduced proteinuria and AGE levels in CKD.
AT1R antagonists [152,153]	They inhibit ROS and exert anti-inflammatory effects by reducing angiotensin II signaling.	Already used in clinical practice for hypertension. In diabetes and hypertension, a significant decrease in AGE levels was demonstrated with valsartan and candesartan, which also reduced albumin excretion.
Metformin [154,155]	It controls glucose homeostasis, reduces gluconeogenesis, and traps reactive carbonyl groups.	Already used in clinical practice for diabetes. Research on the anti-AGE effects of metformin mainly focuses on cellular experiments.
SGLT-2 inhibitors [156]	They control glucose homeostasis.	Already used in clinical practice for diabetes. Inhibition of oxidative, inflammatory, and fibrotic reactions in the kidney of diabetic rats, partly via suppression of the AGE–RAGE axis, was described.
sRAGE [157,158,159]	It blocks AGE from binding to RAGE, thereby working as a decoy receptor.	No human data on this agent have been published. Research on sRAGE effects mainly focuses on preclinical models.
Thiazolidinediones [160,161]	They control glucose homeostasis.	An increase in sRAGE levels was observed in clinical trials with pioglitazone and rosiglitazione in diabetes.

ACE, angiotensin-converting enzyme; AGEs, advanced glycation end products; AT1R, angiotensin II receptor type 1; CKD, chronic kidney disease; RAGE, receptor for advanced glycation end products; ROS: reactive oxygen species; SGLT-2, sodium/glucose cotransporter-2; sRAGE, soluble receptor for advanced glycation end products.

Most of clinical data available on the use of anti-AGE compounds have been obtained in DM cohorts. Some of these studies looked at kidney damage as a DM complication. Only a few of studies have been performed in CKD patients; however, these studies only focused on CKD progression and not on other CKD-related complications such as vascular remodeling and heart failure. The need for studies in this field is therefore compelling.

## 8. Methods for AGE Quantification

As previously discussed, AGEs have been suggested as potential biomarkers in many diseases, but their use in clinical practice has not been implemented to date. Analytical approaches that are currently used in the research field range from global AGE quantification to evaluation of specific compounds. The real challenge associated with introducing their use in clinical practice is the development of robust and standardized analytical methods. The main methods described in the literature include enzyme-linked immunosorbent assays (ELISAs), measurement of skin autofluorescence (SAF) or fluorescent AGEs on other biological samples such plasma and serum, and more sophisticated techniques such as mass spectrometry (MS)-based analyses. SAF and quantification of fluorescent AGEs by measuring the fluorescence properties shared by different compounds allow for the quantification of a specific class of AGEs, including pentosidine. ELISAs and MS offer the possibility of measuring single targets, such as CML or methylglyoxal-hydroimidazolone-1 (MG-H1), or more AGEs simultaneously [162,163].

In their review on analytical methods for assessing AGEs, Jaisson S. et al. indicated ELISA and SAF as the most widely used methods for total AGE quantification (60% of the 70 articles evaluated for their manuscript). The most frequently measured individual AGE was CML (in 20% of articles). Less than 10% of studies focused on other targets, such as pentosidine, carboxyethyl lysine (CEL), MG-H1, argpyrimidine, and glucosepane. ELISA was the most used method for individual AGE quantification. The main differences between methods deal concern the possibility of measuring individual or multiple compounds, equipment availability, and costs. MS analysis is highly promising for AGE detection, but it is time-intensive and costly. In recent years, novel in vivo and non-invasive spectroscopic methods that measure the autofluorescence of AGEs have been developed, and the quantification of skin autofluorescence as a marker of AGE accumulation has become a non-invasive method for use in clinical practice [163]. However, detection of the autofluorescence of AGEs is limited because it can exclusively measure the total fluorescent glycation. The same detection method can be used on plasma. On one hand, this method cannot quantify total AGEs or identify the contribution of individual compounds as pathogenetic molecules and/or biomarkers; on the other hand, it easily allows for the quantification of multiple compounds that can provide more information about AGE production. From a technical point of view, autofluorescence detection is limited by expression in arbitrary units and a lack of standardization. To date, the lack of standardization represents the main limitation of clinical application of AGE assays. Moreover, since the characterization of new AGEs is still ongoing, new appropriate assays need to be developed. With respect to SAF assessment, it would be also necessary to transform arbitrary units into AGE concentration equivalents.

## 9. Conclusions

AGE accumulation is a naturally occurring aging process that is further promoted in conditions that promote AGE synthesis and reduced detoxification, such as CKD. AGEs have a pathogenetic role since they can activate responses leading to the progression of CKD and CKD-related disorders. AGEs, along with different forms of sRAGE, can work as circulating biomarkers for CKD risk stratification. Collectively, the studies discussed in the present review seem to suggest that targeting the AGE–RAGE pathways could be useful in preventing the damaging effects of these molecules on different organs.

## Data Availability

Not applicable.
